# Effects of different intensities of repetitive peripheral magnetic stimulation on corticospinal excitability and motor performance in healthy humans

**DOI:** 10.1371/journal.pone.0349300

**Published:** 2026-05-22

**Authors:** Ayu Omiya, Kanau Shitara, Dai Miyazaki, Mitsuhiro Nito

**Affiliations:** 1 Department of Occupational Therapy, Yamagata Prefectural University of Health Sciences, Yamagata, Japan; 2 Graduate School of Health Sciences, Yamagata Prefectural University of Health Sciences, Yamagata, Japan; Beijing Sport University, CHINA

## Abstract

Repetitive peripheral magnetic stimulation (rPMS) has been applied in clinical settings to enhance the recovery of motor function following central nervous system lesions. However, the optimal intensity of rPMS for inducing neural plasticity and the mechanisms behind its action are not understood. We investigated the impacts of rPMS at two different stimulus intensities on motor performance and the motor-evoked potentials (MEPs) elicited by transcranial magnetic stimulation in the arm muscles of 20 healthy adults. The biceps brachii (BB) muscle was subjected to rPMS in a 2-s ON and 2-s OFF cycle (for a total duration of 15 min). Two levels of rPMS were used: one that was sufficient to cause muscle contraction and one that was not. When investigating the effects on motor performance, an increase in elbow flexion torque and muscle activity was observed after rPMS at an intensity that elicited muscle contraction, whereas no significant changes were observed after rPMS at an intensity that did not cause muscle contraction. The MEPs of the BB increased after rPMS at an intensity that elicited muscle contraction, but no significant changes were observed after rPMS at an intensity that did not cause muscle contraction. Cervicomedullary MEP elicited by transmastoid electrical stimulation did not change after rPMS, implying that the increase in MEP was not accompanied by changes in the efficacy of cortico-motoneuronal synaptic transmission. These findings suggest that rPMS-induced muscle contraction can increase corticospinal excitability while maintaining spinal motoneuron excitability, thereby improving motor performance.

## Introduction

Repetitive peripheral magnetic stimulation (rPMS) has the potential to enhance recovery from motor dysfunction following central nervous system lesions (CNS) by enabling the contraction of paralyzed muscles without causing pain [1–5]. An increase in corticospinal excitability caused by peripheral nerve excitation through rPMS played a crucial role in facilitating motor output and promoting functional recovery [1,6,7].

Stimulus intensity is a crucial factor in application of rPMS for improving movement disorders following CNS. Previous investigations have reported using rPMS intensities that produce palpable muscle contractions or small joint movements [1,3,4,7–10], and maximal joint movements [11]. Further, some studies have applied a predetermined percentage of the maximal stimulator output without considering the response of the target muscle [12–15]. Based on these studies, rPMS at an intensity that induces muscle contractions appears to be effective in improving movement disorders following CNS. These effects are likely due to the activation of low-threshold afferents, such as Ia afferents from muscle spindles, Ib afferents from Golgi tendon organs, and cutaneous afferents [16–18]. Interestingly, previous studies using electrical stimulation have reported that corticospinal excitability was increased when the stimulation intensity was sufficient to induce muscle contraction, but decreased at subthreshold intensity [19–21], suggesting that stimulation intensity may have differential effects on corticospinal excitability. Although similar mechanisms might be expected with magnetic stimulation, the recruitment patterns of afferent and efferent fibers appear to differ from those observed with electrical stimulation. In general, electrical stimulation with increasing strength elicits action potentials in the largest fibers first, as they have the lowest electrical resistance, followed by progressively smaller fibers [16]. In contrast, it has been reported that when magnetic stimulation is applied to peripheral nerves, unlike electrical stimulation, the excitation threshold of sensory fibers is higher than that of alpha motor fibers [22]. In addition, since the eddy currents induced by magnetic stimulation directly stimulate deep tissues without penetrating the skin, it is assumed that the cutaneous afferents are barely activated [2]. Thus, we hypothesize that suprathreshold rPMS, which induces muscle contractions and activates low-threshold afferents, will enhance both motor performance and corticospinal excitability. In contrast, subthreshold rPMS, which does not elicit muscle contractions, may fail to activate low-threshold afferents and will not result in such enhancements. Investigating these differential effects of suprathreshold and subthreshold rPMS may provide insights into stimulation intensity-dependent mechanisms and contribute to optimizing rPMS for various clinical settings.

Further, the underlying mechanisms by which rPMS affects the central nervous system remain unclear. Previous studies demonstrated that rPMS increases the motor-evoked potentials (MEPs) elicited using transcranial magnetic stimulation, likely through a decrease in the short-latency intracortical inhibition and an increase in intracortical facilitation [1,6,7]. However, while these findings suggest a cortical origin, the potential contribution of spinal excitability to rPMS-induced effects cannot be entirely ruled out. The lack of change in the Hoffmann-reflex (H-reflex) after rPMS [7,23] does not necessarily imply that motor neuron excitability and Ia afferent input are unaffected. Since the H-reflex is influenced by both factors [17], it remains challenging to determine whether its stability truly reflects a lack of modulation in these underlying mechanisms. To address this, we employed cervicomedullary MEPs (CMEPs) elicited by transmastoid electrical stimulation in addition to MEPs. By comparing changes in MEPs and CMEPs, we aimed to further delineate the potential site of neural plasticity—whether cortical or spinal—and to clarify how these responses contribute to the overall enhancement of corticospinal excitability.

## Participants and methods

### Participants

Twenty healthy volunteers (12 females, mean ± standard deviation: 21.9 ± 3.7 years) were recruited between February 2024 and March 2025. None of the participants had a history of neurological disease or were taking any medication that affected the CNS. Sample size was determined based on previous studies that investigated the effect of rPMS on corticospinal excitability [6,7]. The dominant hand of each participant was identified using Chapman’s dominant hand test [24], and all participants except one were right-handed. The experimental procedures were approved by the Ethics Committee of Yamagata Prefectural University of Health Sciences (Approval number: 2308−15) following the Declaration of Helsinki. All participants gave written informed consent before the experimental procedures.

### Electromyographic recordings

Electromyographic (EMG) signals were recorded using a pair of 0.8 cm diameter Ag/AgCI disk surface electrodes (NT-211, Nihon Kohden, Tokyo, Japan). The electrodes were attached to the skin overlying the muscle belly of the right biceps brachii (BB) and triceps brachii (TB) with a 2-cm interelectrode distance. To ensure identical placement across sessions, we carefully positioned the electrodes using anatomical landmarks (e.g., the medial epicondyle of the humerus) and individual skin features. Before attaching the electrodes, the skin was rubbed with an alcohol pad to reduce the impedance between the electrodes. The EMG signals were amplified, filtered (15–1,000 Hz), and sampled at 2,000 Hz for offline analysis (Micro 1401 with Signal software, Cambridge Electronic Design, Cambridge, UK).

### Repetitive peripheral magnetic stimulation

A biphasic pulse of rPMS was delivered through a magnetic stimulator (Pathleader, IF-PL1001, IFG, Sendai, Japan). During the rPMS, participants placed their right upper limb on the table with the shoulder 90° flexed, the elbow flexed 0°–10°, and the forearm supinated. The coil was placed on the skin over the right BB muscle. The stimulation site was the muscle belly of the right BB, where BB contraction was achieved at low intensity using rPMS. This posture was selected to ensure the stability of the coil placement and to minimize limb displacement by the rPMS-induced muscle contraction. According to a previous report [7], rPMS was delivered at 25 Hz in a 2 s ON and 2 s OFF cycle for 15 minutes, totaling 225 cycles. The stimulus intensity was set to two conditions: an intensity that could induce muscle contraction (1.2 times the motor threshold, 1.2 × MT), which was based on the protocol of a previous report [7], and that could not induce any muscle contraction (0.8 times the motor threshold, 0.8 × MT), which served as a control condition to minimize the recruitment of group I afferents [25]. The MT was established as the lowest intensity at which muscle contraction could be palpated during rPMS and confirmed by at least two experimenters, and was expressed as a percentage of the maximum stimulator output (%MSO).

### Motor performance

**Fig 1:**
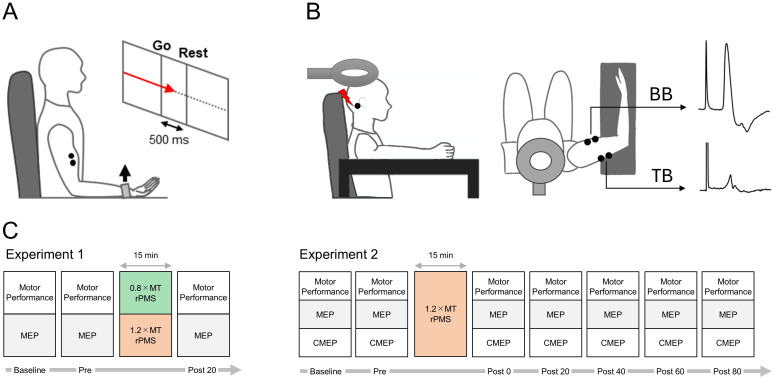
Experimental setup. A: Motor performance measurements. A visual go cue (red arrow) was displayed in front of the participants, who then performed an isometric maximal voluntary elbow flexion. B: Motor-evoked potential measurements. During testing, the upper limb was placed on the stand, and the electromyographic (EMG) activity was recorded from the biceps brachii (BB) and triceps brachii (TB) muscles. C: Schematic representation of the experimental protocols.

Motor performance was assessed through ballistic movements, which were assumed to be sensitive to changes in corticospinal excitability [26-28]. Participants were comfortably seated, with the examined right arm resting on an armrest, the shoulder flexed 45°–60°, the elbow flexed 30°–45°, and the forearm supinated (Fig 1A). A strain gauge (9E01-L31, Sanei, Tokyo, Japan) was placed on the distal forearm using a steel frame to measure elbow flexion torque, with care taken to avoid straddling the wrist. The position of the strain gauge was marked with a pen to ensure consistent measurements throughout the experiment. Two vertical lines, representing a 500 ms time frame, were displayed in front of the participant. The cursor moves from left to right on the horizontal line in the display, and participants were instructed to conduct isometric maximum voluntary elbow flexion as quickly as possible when the cursor reaches the first vertical line and to return to the resting position before it reaches the second vertical line. We took care to prevent any compensatory movements by stabilizing the wrist, shoulder, and trunk throughout the measurement. During the measurement, participants did not receive any visual feedback on their motor performance. Elbow flexion was measured 10 times at each time point, with approximately a 30-s interval between each measurement. To minimize the influence of the initial familiarization effect, the first three trials were excluded from the analysis. The peak torque of the remaining seven trials was averaged and expressed relative to the first baseline measurement. Additionally, the root mean square (RMS) value of the rectified EMG from the BB muscle during the first 100 ms of EMG onset was calculated. EMG onset was defined as the point when the EMG signal increased by 100 μV above baseline.

### Motor-evoked potentials

The participants were comfortably seated, and the examined right arm resting on an armrest with the shoulder flexed at 0°–30° and abducted at ~75°, the elbow flexed 70°–90°, and the forearm supinated as per the previous report [29] (Fig 1B). MEPs were elicited using transcranial magnetic stimulation with a circular coil (loop diameter 90 mm) connected to Magstim 200 (Magstim Company, Whitland, Dyfed, UK). To elicit MEP of the right arm muscles, the stimulus current was applied counterclockwise. The coil was initially placed horizontally over the participant’s vertex, and the ideal location to elicit an MEP in the right BB and TB muscles at rest (hot spot) was determined by adjusting the coil position. The hot spot was defined as the region where the highest MEP of the BB and the lowest stimulation intensity, and the coil position was marked on the scalp with a pen so that it could be kept consistent throughout the experiment. The stimulus intensity was set at 1.2 times the resting MT, which was defined as the minimum intensity at which more than 50% of the MEPs with amplitudes greater than 0.05 mV were elicited. The resting MT was expressed as a percentage of the maximum stimulator output (%MSO). The interstimulus interval ranged from 5–6 s. In one session, 15 MEPs were recorded in the resting condition, and the peak-to-peak amplitude of each was analyzed. To confirm the resting state of the target muscle, the RMS value of the pre-stimulus EMG was calculated for a period of 10–60 ms prior to the stimulation.

### Cervicomedullary motor-evoked potentials

CMEPs were elicited by transmastoid electrical stimulation (200 μs duration) using a constant-current stimulator (DS7R, Digitimer, Welwyn Garden City, UK) with surface electrodes attached to the bilateral mastoid processes. To prevent cervical root activation, the anode was positioned on the right side [30]. The stimulus intensity was adjusted so that the CMEP amplitude (mean ± SD: 0.38 ± 0.21 mV for baseline) matched the MEP amplitude (0.28 ± 0.15 mV for baseline in Experiment 2; *p* = 0.086, d = 0.777), with a range of 80–300 mA. This procedure ensured that both MEPs and CMEPs were elicited from a similar proportion of the motoneuron pool, allowing for a direct comparison of excitability changes between the cortical and spinal levels. To ensure that the responses were not contaminated by cervical root activation, we confirmed that the amplitude of CMEPs was markedly increased during elbow flexion compared to rest at the beginning of the experiment. We also confirmed that the onset latency remained constant (7.68 ± 0.98 ms for baseline) for each participant throughout the experiment (F_6, 36_ = 1.285, *p* = 0.289, $\overset{2}{\underset{p}{\eta }}$ = 0.176). The interstimulus interval was set at 5 s. Ten CMEPs were recorded in the resting condition, and the peak-to-peak amplitude of each was analyzed.

### Maximal motor responses

The maximal motor response (M-max) was elicited by electrical stimulation applied to the brachial plexus at the posterior triangle of the neck using a constant-current stimulator (DS8R, Digitimer, Welwyn Garden City, UK) via bipolar surface electrodes (1.0 cm diameter, 1.5 cm interelectrode distance). The stimulus intensity was set at an intensity of 120% to induce M-max. The inter-stimulus interval was 4–5 s.

### Experimental procedures

#### Experiment 1: Effects of different stimulus intensities of rPMS on motor performance and MEPs.

The stimulus intensity of rPMS was set at 1.2 × (36.4 ± 9.9%MSO) and 0.8 × MT (23.8 ± 7.2%MSO). A paired t-test revealed no significant difference between the MT values used for these two rPMS conditions (the mean values for 1.2 × and 0.8 × MT were 30.3 ± 8.2%MSO and 29.8 ± 9.0%MSO, respectively, *p* = 0.101, d = 0.385). Further, the effects of both intensities were examined in all 20 participants on separate days, at least one day apart. Note that MEP data was absent in one participant because BB MEPs could not be elicited using the maximal stimulator output (100%MSO). Motor performance and MEPs were recorded 10 minutes before (Baseline), immediately before (Pre), and after rPMS (Post 20) (Fig 1C). At each time point, the motor performance and MEPs were recorded in a random order across participants to minimize any potential order effects, with a rest interval of at least 3 min between measurements. These measurements were normalized individually by calculating the percentage change at each time point relative to the Baseline value. Each rPMS intervention was carried out on a different day in a random order.

#### Experiment 2: Lasting effect of 1.2 × MT rPMS on motor performance, MEP, and CMEP.

The stimulus intensity of rPMS was set at 1.2 × MT (37.3 ± 9.8%MSO), and the lasting effect of rPMS on motor performance and MEPs was examined in all 20 participants. Seven out of the 20 participants were also examined for the effect of rPMS on CMEPs, because only they could tolerate the pain and discomfort associated with the transmastoid stimulation. Each measurement was conducted 10 min before the rPMS (Baseline), immediately before (Pre), and every 20 min for 80 min after the rPMS (Post 0, 20, 40, 60, 80) (Fig 1C). At each time point, the motor performance, MEPs and CMEPs were recorded in a random order across participants to minimize any potential order effects, with a rest interval of at least 3 min between measurements. These measurements were normalized individually by calculating the percentage change at each period relative to the baseline value. During recording of MEPs and CMEPs, the RMS value of the pre-stimulus EMG (background EMG) was calculated for a period of 10–60 ms prior to the stimulation to confirm the resting state of the target muscle. Further, in 10 out of the 20 participants, M-max was recorded before and immediately after rPMS.

### Data analysis

Normality was confirmed using the Shapiro–Wilk test. Sphericity was tested using Mauchly’s test. When sphericity was not met, the Greenhouse-Geisser correction was used. If data were not normally distributed, a log transformation was performed, and normality was reassessed. If normality was confirmed after transformation, parametric analyses were conducted; otherwise, non-parametric analyses were conducted. In Experiment 1, a paired t-test was employed to compare the MT in the two rPMS and the baseline values of elbow flexion torque and MEPs between the two rPMS conditions. For the baseline EMG values, the Wilcoxon signed-rank test was employed, as the data did not follow a normal distribution. A two-way repeated-measures analysis of variance (ANOVA) was performed using a linear mixed-effects model, with Intensity (1.2 × MT and 0.8 × MT) and Time (Pre and Post 20) as within-participant factors to compare the elbow flexion torque, EMG, and MEPs. The smallest detectable change was calculated from the amount of facilitation in elbow flexion torque, EMG, and MEP amplitude using the following formula: (standard error of measurement) × 1.96 × √ 2 [31]. In this formula, √2 accounts for the variance associated with two independent sessions (e.g., Pre, Post 20) used to calculate the change score, and 1.96 corresponds to the 95% confidence interval, assuming normally distributed data. Participants demonstrating a greater change than this criterion were classified as responders and those who did not exceed were categorized as non-responders. Spearman’s rank correlation coefficients were calculated to assess the associations across the elbow flexion torque, EMG, and MEPs. In Experiment 2, a paired t-test was employed to compare MEPs and CMEPs at baseline. A one-way repeated-measures ANOVA was used to determine the impact of Time on elbow flexion torque, EMG, MEPs, CMEPs, and CMEP latency. A two-way repeated-measures ANOVA was employed using a linear mixed-effects model, with Potentials (MEP and CMEP) and Time (Pre, Post 0, Post 20, Post 40, Post 60, and Post 80) as within-participant factors to compare the amplitudes of the evoked responses and background EMGs. A paired *t*-test with Bonferroni correction was used for all post-hoc comparison, with the exception of the torque data in Experiment 1, for which the Wilcoxon signed-rank test with Bonferroni correction was used. Since TB MEPs could be recorded in only four participants, statistical analysis was not performed.

Effect sizes were reported as partial η² ($\overset{2}{\underset{p}{\eta }}$) for ANOVA, Cohen's d for the t test and, r for the Wilcoxon signed-rank test. The statistical analyses were carried out using SPSS 30 (IBM, Armonk, NY, United States), with a significance level of 5%.

## Results

### Experiment 1: Effects of different stimulus intensities of rPMS

#### Motor performance.

When comparing the baseline values of elbow flexion torque under the two rPMS conditions, the average values of 1.2 × and 0.8 × MT were 67.6 ± 27.9 Nm, and 66.3 ± 28.6 Nm, respectively, with no significant difference (*p* = 0.410, d = 0.189). Comparing the baseline values of EMG under the two rPMS conditions, the average values of 1.2 × and 0.8 × MT were 0.35 ± 0.21 mV and 0.38 ± 0.21 mV respectively, with no significant difference (*p* = 0.167, r = 0.037).

**Fig 2:**
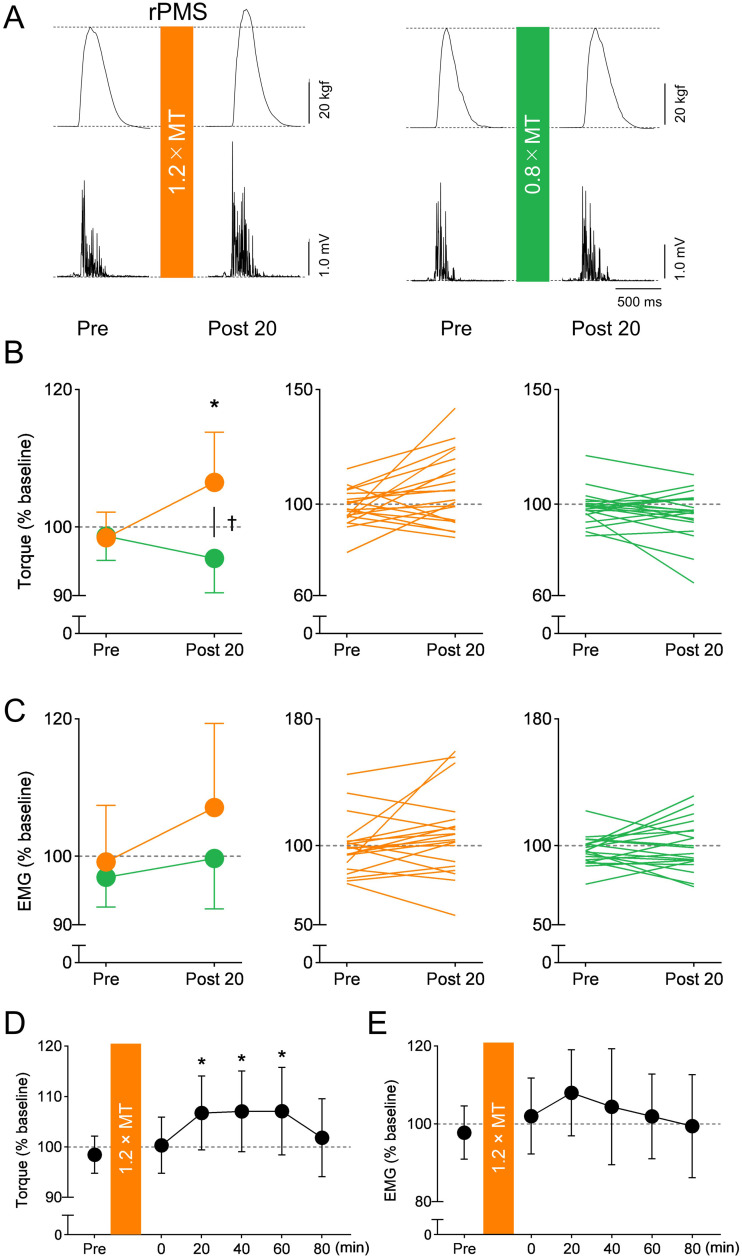
Impacts of repetitive peripheral magnetic stimulation (rPMS) on motor performance. A: Representative waveforms of elbow flexion torque and electromyography (EMG) before and after rPMS recorded from the biceps brachii (BB) muscle of a single participant. B, C: Group averages in elbow flexion peak torque (B) and EMG (C) for the 1.2 × MT (orange) and 0.8 × MT (green) rPMS conditions are shown. D, E: Lasting effects of rPMS on elbow flexion torque (D) and EMG (E). The elbow flexion torque and EMG were normalized at the baseline value. The mean values and 95% confidence intervals obtained from 20 participants are shown. Asterisks show a significant difference compared to “Pre” within each condition, and the dagger shows a significant difference between the 1.2 × MT and 0.8 × MT conditions (*p* < 0.05).

A repeated-measures ANOVA conducted on elbow flexion torque revealed a significant interaction between Intensity and Time (F _1,57_ = 6.048, *p* = 0.017, $\overset{2}{\underset{p}{\eta }}$ = 0.318) and the main effect of Intensity (F _1,57_ = 5.615, *p* = 0.021, $\overset{2}{\underset{p}{\eta }}$ = 0.062), but the main effect of Time was not significant (F _1,57_ = 1.096, *p* = 0.300, $\overset{2}{\underset{p}{\eta }}$ = 0.170). Post-hoc test revealed significantly increased elbow flexion torque after 1.2 × MT rPMS (*p* = 0.037, r = 0.467) but no significant difference after 0.8 × MT rPMS (*p* = 0.167, r = 0.309) (Fig 2B). The comparison of the elbow flexion torque between the 1.2 × MT and 0.8 × MT conditions revealed no significant difference at Pre (*p* = 0.940, r = 0.017) but exhibited a significant difference at Post 20, with higher torque in rPMS at 1.2 × MT (*p* = 0.017, r = 0.534).

A repeated-measures ANOVA conducted on EMG activity revealed no significant main effects of Intensity (F _1,57_ = 1.200, *p* = 0.278, $\overset{2}{\underset{p}{\eta }}$ = 0.035), Time (F _1,57_ = 2.564, *p* = 0.115, $\overset{2}{\underset{p}{\eta }}$ = 0.157) or their interaction (F _1,57_ = 0.773, *p* = 0.383, $\overset{2}{\underset{p}{\eta }}$ = 0.069).

**Fig 3:**
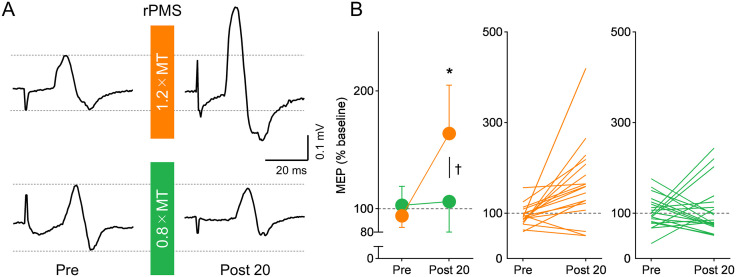
Impacts of repetitive peripheral magnetic stimulation (rPMS) on motor-evoked potentials (MEPs). A: Representative MEP traces recorded from the biceps brachii (BB) muscle of a single participant before and after rPMS. Each waveform represents the average of 15 trials. B: Group averages in MEPs for the 1.2 × MT (orange) and 0.8 × MT (green) rPMS conditions are shown. Asterisk shows a significant difference compared to “Pre” within each condition, and the dagger shows a significant difference between the 1.2 × MT and 0.8 × MT conditions (*p* < 0.05).

***MEPs.*** The experiment involved 19 out of the 20 participants for whom MEPs could be recorded. MEP amplitudes at baseline were comparable between the 1.2 × MT and 0.8 × MT conditions (0.23 ± 0.10 mV and 0.37 ± 0.35 mV, respectively), with no significant difference (*p* = 0.081, d = 0.422). A repeated-measures ANOVA conducted on BB MEPs revealed a significant interaction between Intensity and Time (F_1, 54_ = 6.525, *p* = 0.013, $\overset{2}{\underset{p}{\eta }}$ = 0.079) and the main effects of Intensity (F_1, 54_ = 4.828, *p* = 0.032, $\overset{2}{\underset{p}{\eta }}$ = 0.060) and Time (F_1, 54_ = 8.921, *p* = 0.004, $\overset{2}{\underset{p}{\eta }}$ = 0.105). Post-hoc tests revealed significantly increased BB MEPs after 1.2 × MT rPMS (*p* = 0.002, d = 0.830) but with no significant difference after the 0.8 × MT rPMS (*p* = 0.657, d = 0.118). The comparison of MEPs between the 1.2 × MT and 0.8 × MT conditions revealed no significant difference at Pre (*p* = 0.646, d = 0.125), but a significant difference was observed at Post 20, with higher MEP amplitude in 1.2 × MT rPMS (*p* = 0.021, d = 0.569) (Fig 3).

TB MEPs were able to record in 4 of 19 participants. The average MEP amplitudes before and after rPMS were 0.15 ± 0.03 mV and 0.17 ± 0.03 mV with the stimulus intensity of 1.2 × MT and 0.14 ± 0.04 mV and 0.14 ± 0.05 mV with the stimulus intensity of 0.8 × MT, respectively. No changes were observed in TB MEPs under the two rPMS conditions.

**Fig 4:**
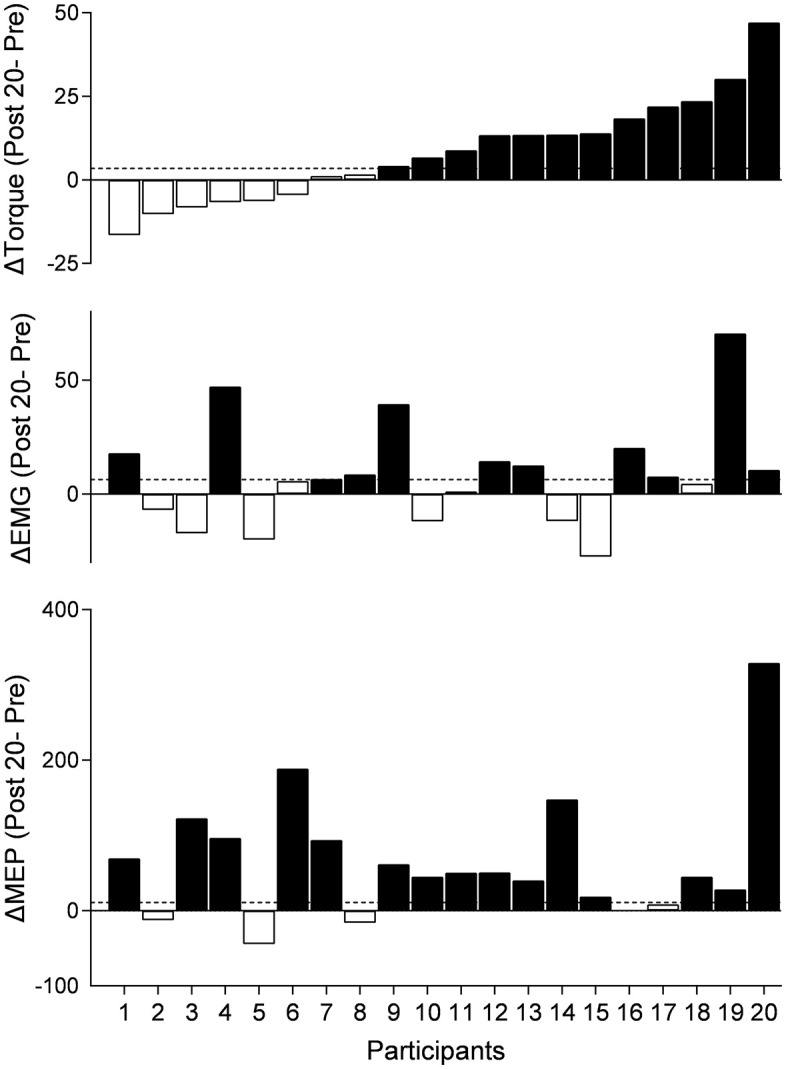
Individual changes in elbow flexion torque, electromyographic (EMG) activity, and motor-evoked potentials (MEPs) after repetitive peripheral magnetic stimulation at 1.2 × MT. Participants were classified as responders (closed bars) or non-responders (open bars) based on the amount of facilitation exceeding the smallest detectable change (horizontal dotted line) in elbow flexion torque, EMG activity, and MEP amplitude recorded from the biceps brachii muscle. Note that MEP data for participant 16 were not available because MEPs could not be elicited even at 100% stimulator output.

***Classifications.*** Participants were classified as responders or non-responders based on the amount of facilitation exceeding the smallest detectable change in elbow flexion torque, EMG activity, and MEP amplitude of BB (Fig 4). For the elbow flexion torque, 12 (60%) of 20 participants were classified as rPMS responders. We classified 10 out of 11 participants within the responders based on the change in elbow flexion torque as responders according to MEP changes (one participant was excluded because MEPs could not be recorded). Further, 7 of 12 participants were also classified as responders based on changes in EMG activity. Among 8 participants classified as non-responders based on elbow flexion torque changes, increases in MEPs and EMG activity were observed in 5 participants each. We found that no significant correlations between any pairwise combination of changes in elbow flexion torque, EMG activity, and MEPs (torque vs EMG, *p* = 0.070, ρ = 0.425; torque vs MEPs, *p* = 0.357, ρ = 0.224; MEPs vs EMG, *p* = 0.991, ρ = 0.003). These findings suggest that an increase in corticospinal excitability may contribute to enhancing elbow flexion torque after rPMS.

### Experiment 2: Lasting effect of 1.2 × MT rPMS

***Motor Performance.*** A repeated-measures ANOVA was carried out to investigate the effect of rPMS on elbow flexion torque and EMG at a stimulus intensity of 1.2 × MT (Figs 2D and E). The results revealed a significant main effect of Time on elbow flexion torque (F_2.65, 50.38_ = 3.009, *p* = 0.045, $\overset{2}{\underset{p}{\eta }}$ = 0.137). Post-hoc test revealed that elbow flexion torque significantly increased at Post 20 (*p* = 0.035, d = 0.508) and 40 (*p* = 0.040, d = 0.492) compared to Pre, whereas no significant changes were observed at other time points (Post 0, *p* = 0.490, d = 0.157; Post 60, *p* = 0.055, d = 0.457; Post 80, *p* = 0.516, d = 0.148). The EMG showed a similar trend to elbow flexion torque, but no significant main effect was observed (F_3.23, 61.28_ = 0.993, *p* = 0.406, $\overset{2}{\underset{p}{\eta }}$ = 0.050).

**Fig 5:**
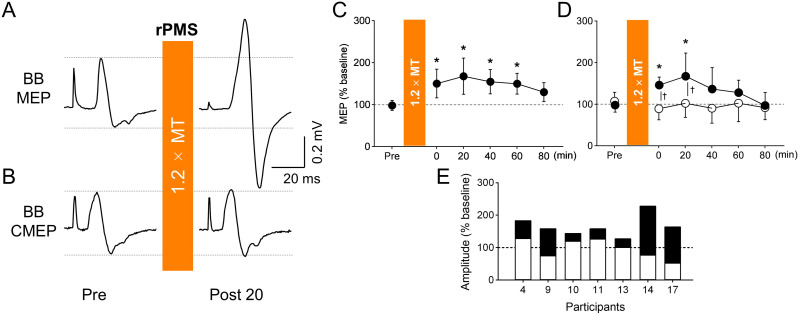
Impact of repetitive peripheral magnetic stimulation (rPMS) on motor-evoked potentials (MEPs) and cervicomedullary MEPs (CMEPs). A, B: Representative waveforms of MEPs (A) and CMEPs (B) recorded from the biceps brachii (BB) muscle of a single participant before and after rPMS. Each waveform represents the average of 15 trials. C: Lasting effects of rPMS on MEPs for all participants (C, n = 19), and both MEPs and CMEPs for the participants in whom CMEPs were recorded (D, n = 7). MEPs and CMEPs were normalized at baseline values. The mean values and 95% confidence intervals are shown. Asterisks show a significant difference compared to “Pre”. E: Individual changes in MEPs (black bar) and CMEPs (white bar) at Post 20 after rPMS. Individual data is normalized to baseline values. Participant numbers correspond to those presented in Fig 4.

***MEP and CMEPs*.** The amplitude of the MEP at baseline was 0.25 ± 0.11 mV in 19 participants. To investigate the lasting effect of rPMS on MEP at a stimulus intensity of 1.2 × MT, a repeated-measures ANOVA revealed a significant main effect of Time for MEP (F_5, 90_ = 6.067, *p* < 0.001, $\overset{2}{\underset{p}{\eta }}$ = 0.252) (Fig 5). Post-hoc test showed that MEP significantly increased at the time points of Post 0 (*p* = 0.003, d = 0.940), 20 (*p* = 0.009, d = 0.844), 40 (*p* = 0.001, d = 1.039), and 60 (*p* < 0.001, d = 1.141) compared to Pre, whereas no significant change was observed at that of Post 80 (*p* = 0.168, d = 0.527).

To compare the effects of rPMS on MEPs and CMEPs in 7 participants, we first confirmed that there were no significant differences in background EMG levels across all time points or between potentials. A two-way repeated-measures ANOVA using a linear mixed-effects model revealed no significant main effects of Potentials (F_1,6_ = 1.148, *p* = 0.325, $\overset{2}{\underset{p}{\eta }}$ = 0.161), Time (F_6,36_ = 0.876, *p* = 0.522, $\overset{2}{\underset{p}{\eta }}$ = 0.127), or their interaction (F_6,36_ = 0.554, *p* = 0.764, $\overset{2}{\underset{p}{\eta }}$ = 0.084) on background EMGs. In contrast, the analysis performed on the MEP and CMEP amplitudes revealed significant main effects of Potentials (F_1,66_ = 23.058, *p* < 0.001, $\overset{2}{\underset{p}{\eta }}$ = 0.454), Time (F_5,66_ = 2.018, *p* = 0.087, $\overset{2}{\underset{p}{\eta }}$ = 0.336), and their interaction (F_5,66_ = 2.868, *p* = 0.021, $\overset{2}{\underset{p}{\eta }}$ = 0.439). Post-hoc test showed that MEP significantly increased at the time points of Post 0 (*p* < 0.001, d = 3.240), and 20 (*p* = 0.046, d = 1.425) compared to Pre, whereas no significant change was observed at any other time points (*p* > 0.05). In contrast, there was no significant main effect of Time for CMEPs (F_1.977, 11.864_ = 0.601, *p* = 0.562, $\overset{2}{\underset{p}{\eta }}$ = 0.091).

***M-max*.** The M-max amplitude before and after rPMS were 8.36 ± 3.91 mV and 8.21 ± 3.62, respectively, and no significant difference was observed between them (*p* = 0.627, d = 0.159).

## Discussion

This study showed that rPMS at a stimulus intensity of 1.2 × MT, but not 0.8 × MT, improved motor performance and increased MEPs but not CMEPs. This suggests that increased excitability of the primary motor cortex, rather than subcortical regions, may contribute to enhanced motor performance.

This study found that 1.2 × MT rPMS improved motor performance and corticospinal excitability, most likely due to the activation of low-threshold afferents. The excitability threshold for electrical stimulation of peripheral nerves is inversely proportional to nerve fiber diameter. Nerves become excited in the following order, from the lowest threshold: Ia fibers from muscle spindles, Ib fibers from Golgi tendon organs, and α motor fibers [16–18]. Further, since rPMS was applied transcutaneously, it is assumed that cutaneous afferents beneath the stimulation site may also be activated. However, the eddy currents induced by magnetic stimulation directly activate deep tissues without penetrating the skin [2]. Thus, the contribution of cutaneous nerves at the stimulation site appears to be minimal or negligible. Instead, the observed enhancements were likely mediated by the recruitment of low-threshold group I afferents (particularly Ia afferents), which are known to be activated by rPMS at intensities sufficient to elicit muscle contraction and contribute to the facilitation of corticospinal excitability [25]. Further, the frequency used in the present study (i.e., 25 Hz) mimics the discharge rate of Ia afferents during voluntary movements [32]. Consequently, the suprathreshold rPMS used in our study likely provided physiological-like sensory input via group I afferents, potentially contributing to the enhancement of both motor performance and corticospinal excitability.

In contrast, 0.8 × MT rPMS did not significantly improve motor performance or MEPs. A previous study has reported that sensory nerve action potentials recorded at the digit and the H-reflex, which is induced by monosynaptic excitation from homonymous group Ia afferents, are not induced at intensities lower than the MT when using magnetic stimulation on peripheral nerves [22]. Consistent with this, subthreshold rPMS has been shown to fail in inducing monosynaptic facilitation via group Ia afferents or MEP enhancement [25]. These findings suggest that the stimulus intensity of 0.8 × MT rPMS used in this study insufficient to excite the afferent fibers required to modulate corticospinal excitability. Considering these results, the observed increase in corticospinal excitability may be partly attributed to input from Ia afferents activated by rPMS. However, because the stimulation intensity of rPMS was set based on the presence or absence of muscle contraction, it remains difficult to identify which specific nerves contributed to the enhancement of motor performance or MEPs. Further research is required to examine the impacts of other rPMS stimulation intensities on motor performance and corticospinal excitability.

Previous studies [6,7] have found that 1.2 × MT rPMS leads to an increase in MEP. These studies found that rPMS reduces short-latency intracortical inhibition and increases intracortical facilitation [6,7]. Furthermore, previous studies have reported that the H-reflex remains unchanged after rPMS [7,23]. Because the H-reflex is influenced by the excitability of spinal motor neurons and excitatory inputs from Ia afferents [17], it was difficult to determine whether the absence of change in the H-reflex could explain the lack of change in these two. To address this, the present study employed CMEPs and compared them with MEPs to evaluate the effects of rPMS. We found that MEPs significantly increased, whereas CMEPs remained unchanged following rPMS. CMEPs are thought to be induced by exciting the axons of the corticospinal tract at the level of the cervicomedullary junction, primarily representing the direct monosynaptic component from the motor cortex to spinal motor neurons [33–35]. The lack of significant change in CMEPs suggests that rPMS may not have substantially influenced the fast-conducting descending volleys. Further, consistent with previous reports [7,23,25], the M-max did not show significant changes following rPMS, suggesting that the efficacy of neuromuscular transmission was not altered by rPMS. Therefore, the increase in MEPs observed in this study is likely to reflect changes at the cortical level rather than at the spinal or peripheral levels. However, it should be noted that CMEPs primarily assess the monosynaptic component of the corticospinal tract, therefore, we cannot entirely exclude the possibility that rPMS might influence other subcortical mechanisms, such as the propriospinal pathway or segmental interneurons, which could also contribute to the modulation of motor output [17].

The 1.2 × MT rPMS increased elbow flexion torque and EMG, while 0.8 × MT induced no significant changes in motor performance. These results are consistent with a previous study that demonstrated increased motor output following suprathreshold rPMS applied to the wrist extensor muscles [7]. Given that ballistic movements are considered to be largely pre-programmed [16,26], they require rapid and highly synchronized recruitment of motor units, which is associated with increased corticospinal excitability [28]. Therefore, the enhancement of motor performance observed in this study could be attributed to an increase in cortical excitability, which potentially facilitates synchronized descending volleys and the recruitment of spinal motor neurons [27]. The trend toward increased EMG amplitude might reflect more synchronous activation of motor units, rather than a substantial increase in the total number of recruited units. An alternative explanation is that, although the firing rate of some motor units might increase, it does not necessarily result in an increase in the RMS value [27,36]. Because surface electrodes were used to record EMG activity and the RMS value was used for quantification, the method employed in the present study was not suitable for detecting changes in the inter-spike intervals of single motor units. Thus, further investigations are required to directly examine the effects of rPMS on motor unit behavior.

rPMS can directly stimulate deep tissues without penetrating the skin to induce eddy currents [2], it is thought to be less likely to cause discomfort during stimulation. This feature suggests that rPMS may be a promising therapeutic tool for individuals with movement disorders following CNS lesions. Whereas numerous studies have reported improvements in motor function following rPMS in individuals with CNS lesions [1,3,4,8–11,13–15], the extent to which these improvements are directly related to rPMS-induced increases in corticospinal excitability, as reflected in MEP amplitude, remains unclear. Further studies are needed to determine whether rPMS-induced changes in corticospinal excitability are directly associated with improvements in motor performance and clinical function in individuals with CNS lesions.

## Limitations

This study has several methodological limitations that should be acknowledged when interpreting the findings. First, the sample size was not determined based on a formal a priori power analysis; instead, it was chosen to be comparable to those used in similar previous studies [6,7]. In particular, the sample size for CMEP measurements was even smaller, as stable responses could only be elicited from a subset of participants. Although this was largely due to the discomfort caused by the stimulation intensity, the possibility of selection bias cannot be ruled out. This limited sample size suggests that caution is warranted when generalizing our findings. Second, we did not assess potential changes in peripheral components across all experiments, as the M-max was not consistently recorded. Although we carefully positioned the surface electrodes to ensure consistency across experiments, we cannot entirely rule out the influence of day-to-day fluctuations in peripheral factors, such as the spatial relationship between the electrodes and the underlying muscle. Further, whereas there were no statistically significant differences in baseline MEP values between conditions in Experiment 1, some variability was observed. It should be noted, however, that baseline EMG levels during the maximal voluntary contraction task showed no significant differences between conditions. Nonetheless, normalizing these values to the M-max would have provided clearer information regarding the relative size of the MEPs, which would have strengthened the reliability of our baseline comparisons. Third, the MT used to determine rPMS intensity was defined as the minimum stimulation intensity required to elicit a palpable muscle contraction. This definition can be more challenging to standardize than MT based on EMG activity, potentially raising concerns regarding reproducibility. However, to address this concern, we employed a procedure in which MT was determined independently by at least two experimenters and finalized only when a consensus was reached. Indeed, the MT values for the two rPMS conditions (0.8 × and 1.2 × MT) were comparable across different experimental days, indicating that the determination of MT was reproducible within participants across sessions. Despite its limitations, this pragmatic definition of MT may be advantageous in clinical settings, where simple and feasible criteria for determining rPMS intensity are required.

## Conclusions

rPMS at an intensity sufficient to induce muscle contraction increased corticospinal excitability by activating low-threshold afferents, thus enhancing motor performance. In contrast, rPMS below the motor threshold was insufficient to excite afferents and did not influence corticospinal excitability or motor performance. We also found that rPMS had no significant effect on the efficiency of cortico-motoneuronal synaptic transmission or the excitability of spinal motor neurons, suggesting that increased excitability in motor-related areas of the cortex likely contributed to the increase in the corticospinal excitability.

## Supporting information

S1 Data Dataset. (XLSX)
